# The Association Between Consumption of Foods/Food Groups and the Risk of Overweight/Obesity and Metabolically Unhealthy Obesity in Children and Adolescents: A Systematic Review and Meta-Analysis

**DOI:** 10.3390/life16060934

**Published:** 2026-06-01

**Authors:** Fidelia Bature, Michael Georgoulis, Athanasia Kyrkili, Meropi D. Kontogianni, Zoi-Eleni Koti, Chara Kapsala, Iliana Korma, Yannis Pappas

**Affiliations:** 1 Institute for Health Research, Putteridge Bury Campus, University of Bedfordshire, Luton LU28LE, UK; fidelia.bature@beds.ac.uk; 2 Department of Nutrition and Dietetics, School of Health Sciences and Education, Harokopio University of Athens, 17676 Athens, Greece; mihalis.georgoulis@gmail.com (M.G.); akirkili@hua.gr (A.K.); mkont@hua.gr (M.D.K.); 3 Ukemed Global Ltd. 121, Prodromou Street Offices 713-715, 2064 Nicosia, Cyprus; zeniakoti@ukemed.com (Z.-E.K.); chara.kapsala@ukemedglobal.com (C.K.); i.korma@gmail.com (I.K.)

**Keywords:** foods, food groups, children, overweight, obesity, systematic review

## Abstract

Existing studies have suggested an association between consumption of foods/food groups and the risk of childhood overweight/obesity (OV/OB) and metabolic unhealthy obesity (MUO). However, they are heterogeneous in terms of design, samples and outcomes, and most do not provide evidence of long-term longitudinal associations given their cross-sectional nature. The aim of the present work was to systematically review longitudinal evidence of the association between foods/food groups and the risk of OV/OB and MUO in children and adolescents aged 2–19 years. Two databases (Scopus and PubMed) were searched for original research conducted in Western countries. Prospective epidemiological studies (PES) and randomized controlled trials (RCTs) with exposures/interventions related to the consumption of foods/food groups, OV/OB- or MUO-related outcomes and ≥1-year follow-up were considered eligible. A narrative evidence synthesis, complemented by random-effects meta-analyses where feasible, was performed. The review protocol was registered in PROSPERO (ID: CRD42024496148). The narrative synthesis of 23 longitudinal studies revealed a detrimental effect of sugar-sweetened beverages (SSBs) (n = 8/10 PESs and 1/2 RCTs) and ultra-processed foods (UPFs) (n = 2/3 PESs), and a beneficial effect of full/higher-fat dairy products (n = 2/3 PESs) on OV/OB-related outcomes, although certainty in evidence was (very) low. Evidence was inconclusive for artificially sweetened beverages, fruits and vegetables (primarily 100% fruit juices), milk and total dairy products. Random-effects meta-analysis of PESs focusing on SSBs revealed a positive association with follow-up body mass index (n = 3, pooled beta: 0.16 kg/m^2^, 95%CI: 0.09, 0.23) but a non-significant association with change in BMI (n = 3, pooled beta: 0.07 kg/m^2^, 95%CI: −0.05, 0.19). Only 1 PES reported on MUO-related outcomes and revealed a potential beneficial link between higher-fat milk intake and selected cardiometabolic indices. In conclusion, consumption of SSBs is positively associated with indicators of childhood OV/OB risk. A detrimental effect of UPFs and a beneficial effect of higher-fat dairy products on childhood adiposity outcomes were also observed, but the available evidence remains limited and insufficient to draw robust conclusions. Data for other foods/food groups and OV/OB, as well as for their link with childhood MUO, remain scarce and inconclusive.

## 1. Introduction

The prevalence of childhood overweight/obesity (OV/OB) continues to rise globally. It is estimated that one in three children and adolescents in the European Union have OV or OB [1]. Lifestyle parameters, and specifically diet, have been suggested as important risk factors for OV/OB. Several individual studies have focused on the association between habitual intake of specific foods/food groups and childhood OV/OB-related outcomes with varying findings. Recent systematic reviews and meta-analyses of epidemiological evidence published up until 2022 have revealed that unhealthy foods and beverages, most importantly sugar-sweetened beverages (SSBs) and fast foods, are consistently linked to higher adiposity indices and odds of OV/OB in childhood; however, they have also highlighted a dearth of high-quality evidence from prospective epidemiological studies, a high degree of heterogeneity in study characteristics and inconsistency in effect estimates, as well as the lack of data for other foods/foods groups, especially those considered part of a healthy diet [2,3]. Accordingly, a recent systematic review and meta-analysis of randomized controlled trials (RCTs) suggested that dietary shifts from lower-fat to higher-fat dairy products and from sugar-sweetened to non-caloric beverages can result in favorable changes in body composition parameters among children and adolescents, albeit the evidence synthesis included RCTs with short follow-up periods (2–36 weeks) and no data were identified for other foods/food groups [4].

Along with the rise in OV/OB rates, there has also been a considerable increase in adiposity-related cardiometabolic burden, and metabolically unhealthy obesity (MUO); i.e., OB coupled with metabolic pathology (e.g., dyslipidemia, hypertension or glucose metabolism abnormalities), is an increasing public health concern among children and adolescents. According to recent epidemiological data, the global prevalence of metabolic syndrome is 2.8% in children and 4.8% in adolescents, equating to around 25.8 million children and 35.5 million adolescents living with metabolic syndrome [5]. OB, particularly abdominal adiposity, drives this cardiometabolic burden through excessive adipose tissue that acts as a dysfunctional endocrine organ, promoting insulin resistance, ectopic fat accumulation, chronic low-grade inflammation, and oxidative stress, and ultimately leading to cardiometabolic pathology (hypertension, type 2 diabetes and cardiovascular disease) [6]. The recent systematic review by Markey et al. [7] focused on evidence of the relationship between consumption of foods/food groups and children’s cardiometabolic profile and revealed that a high exposure to unhealthy foods and beverages, specifically UPFs, in early childhood is associated with a detrimental blood lipid and blood pressure profile in later childhood. However, the findings of this work are related to individual cardiometabolic risk biomarkers and are not specific to the MUO phenotype (presence of OB coupled with cardiometabolic disorders), while the review also highlighted a high degree of methodological heterogeneity and a low quality of evidence coming from the few included prospective cohort studies published up to 2022 [7].

Although previous systematic reviews and meta-analyses have explored the association between consumption of specific foods/food groups and adiposity in childhood and adolescence, their findings are limited by the predominance of cross-sectional studies in evidence synthesis, the scarcity of high-quality longitudinal studies, the short duration and follow-up of clinical trials, the limited investigation of foods/food groups considered part of a healthy diet, the insufficient assessment of MUO-related outcomes, and the lack of incorporation of the most recent literature in this rapidly evolving field [2,3,4,7]. Therefore, the aim of the present systematic review and meta-analysis was to evaluate the association between consumption of foods/food groups and indicators of childhood OV/OB and MUO risk, focusing only on data from long-term studies with a longitudinal design. A comprehensive and up-to-date synthesis of the current longitudinal evidence can offer valuable insight into the role of diet—at the level of individual foods and food groups—in the development of adiposity and its cardiometabolic manifestations, and support the design of effective strategies for promoting healthy dietary habits, healthy body weight and optimal metabolic health among children and adolescents.

## 2. Materials and Methods

### 2.1. Research Objective and Questions

A systematic review and meta-analysis was performed to collect and synthesize evidence regarding the association between food/food group consumption and the risk of childhood OV/OB and MUO, according to the Preferred Reporting Items for Systematic reviews and Meta-analyses (PRISMA) guidelines [8,9] (the PRISMA 2020 Checklist is available in Supplementary Table S1 [10]). The systematic review protocol was registered in PROSPERO (registration number: CRD42024496148). The research questions were formulated according to the Population-Exposure-Comparator-Outcome (PECO) model for epidemiological studies and the Population-Intervention-Comparator-Outcome (PICO) model for interventional studies. The population of interest was children and adolescents 2–19 years of age. The systematic review focused on epidemiological studies with exposures relevant to foods or food groups (high/low consumption versus low/high consumption) and interventional studies aiming to increase/reduce the intake of specific foods or food groups (versus standard care or the absence of intervention). The outcomes of interest were the presence of OV/OB and MUO (OB coupled with cardiometabolic disorders), as well as individual indicators of OV/OB (e.g., body mass index, waist circumference, fat mass, fat-free/lean mass) and MUO (e.g., lipidemic profile, blood pressure, glucose metabolism indices, inflammatory markers).

### 2.2. Eligibility Criteria

Original peer-reviewed journal articles published in English were eligible for inclusion in the systematic review. The search timeframe was set to cover a period of 10 years before the registration of the systematic review protocol in PROSPERO, i.e., from 1 January 2013 until 30 June 2024. This timeframe was chosen to facilitate a comprehensive coverage of the relevant scientific literature in a timely and efficient manner, taking into consideration the big volume of evidence and the rapid advancements in the field of nutrition and childhood OB, and to ensure the synthesis of studies with solid scientific background, high methodological quality and transparent reporting that reflect current standards in nutritional epidemiology and childhood OB diagnosis, assessment and prevention. In terms of study design, prospective epidemiological studies and RCTs exploring longitudinal associations between foods/food groups and OV/OB- or MUO-related outcomes were considered eligible. A follow-up of ≥12 months was also set as an inclusion criterion for both study designs, as this timeframe was deemed adequate to observe meaningful changes in the outcomes of interest. The geographical regions of interest were Western countries [i.e., countries in Europe, North America (USA, Canada) and Oceania]. Western countries exhibit some of the highest prevalence rates of childhood OV/OB globally, and share common obesogenic environmental/cultural characteristics and similar public health and policy action agendas [1]. Targeted research in these settings is important to limit heterogeneity and enhance comparability among studies, given the significant socio-cultural and environmental variability (dietary norms and food environments) compared to non-Western countries, and produce relevant outcomes for public health and decision-making.

Based on the formulated research questions, studies in animals, those with a population of children under 2 years of age or adults above 19 years of age, those focusing on exposures/interventions not related to foods/food groups (including other types of dietary exposures, such as nutrients, dietary patterns and meal patterns), and those focusing on outcomes not relevant to OV/OB or MUO, were excluded as non-relevant. Additionally, studies with other methodological designs (e.g., cross-sectional studies, case–control studies and non-randomized uncontrolled trials), those conducted in non-Western settings (South America, Asia and Africa), as well as those with a <12-month follow-up period, were also excluded.

### 2.3. Literature Search and Study Selection

Literature search was carried out in Scopus and PubMed using a combination of free-text terms relevant to population (children and adolescents), exposures/interventions (foods and food groups), outcomes (OV/OB and MUO), and study design. The exact search query can be found in Supplementary Table S2. A manual search of the reference lists of included studies and previous systematic reviews was also performed to enhance search comprehensiveness. The identified studies were subsequently imported into the Zotero software (version 7.0), duplicates were removed and unique reports was screened for eligibility in two phases. Firstly, two researchers (FB and ZK) independently examined the titles and abstracts to identify eligible reports for full retrieval. A third senior researcher (YP) re-examined the first scan for consolidation. Secondly, after exclusions based on titles/abstracts, the full-text articles of the remaining studies were retrieved, and their eligibility was evaluated independently by two researchers (FB and ZK) for the final study selection. Any disagreements were resolved by a third senior researcher (YP).

### 2.4. Risk of Bias Assessment

Prospective epidemiological studies were evaluated for risk of bias using the Risk of Bias in Non-randomized Studies of Exposures (ROBINS-E) tool [11]. The tool assesses bias in 7 domains, including (1) confounding; (2) measurement of the exposure; (3) selection of participants into the study (or into the analysis); (4) post-exposure interventions; (5) missing data; (6) measurement of the outcome; and (7) selection of the reported result. Similarly, RCTs were assessed for risk of bias using the revised tool for Risk of Bias in randomized trials (ROB-2), which is structured into 5 domains, focusing on different aspects of trial design, conduct and reporting: (1) randomization process; (2) deviations from intended interventions; (3) missing outcome data; (4) measurement of the outcome; and (5) selection of the reported result [12]. Based on the above-mentioned tools, studies were characterized as “low risk”, “some concerns”, “high risk” or “very high risk” in terms of risk of bias.

### 2.5. Data Extraction and Synthesis

Data from the eligible studies were extracted from full texts by one researcher (FB), with a second researcher (YP) randomly rechecking the process. These included information about general study characteristics (e.g., country, setting, duration), population characteristics (sample size, age, sex), exposures (type, definition, assessment), interventions (duration, delivery, groups, content), outcomes (type, definition, assessment), statistical analysis (tests, confounders), and main results. Studies were grouped and qualitatively synthesized according to the exposure/intervention (food or food group of interest). Evidence was narratively synthesized based on the Synthesis Without Meta-analysis (SWiM) guidelines [13]. Synthesis was performed separately for OV/OB- and MUO-related outcomes, and within each outcome category separately for epidemiological studies and RCTs. Within each subcategory, studies with common exposures/interventions in terms of foods/food groups were qualitatively synthesized based on the direction and significance of the observed associations/effects.

When feasible, a random-effects meta-analysis of studies with common exposures/interventions and outcomes was performed per methodological design (separately for prospective epidemiological studies and RCTs) using Der Simonian and Laird’s method [14]. Effect estimates from adjusted models exploring the effect of foods/food groups on indicators of childhood OV/OB or MUO risk were extracted from the eligible studies. Combined estimates and their 95% confidence intervals (CI) were obtained using inverse-variance weighting. Heterogeneity across studies was evaluated by the Cochran Q and I^2^ [15,16]. I^2^ values of 0–25%, 25–50%, 50–75% and >75% were considered indicative of low, low-to-moderate, moderate-to-high and high heterogeneity, respectively. The stability of results was assessed through a leave-one-out sensitivity meta-analysis, in which each study was removed at a time and pooled estimates were re-calculated. All analyses were performed in STATA version 18 (StataCorp, College Station, TX, USA) and statistical significance was set at *p* < 0.05.

### 2.6. Certainty of Evidence

The Grading of Recommendation, Assessment, Development and Evaluation (GRADE) system was used to evaluate the certainty of evidence for the effect of exposures/interventions on the outcomes of interest [17]. Each evidence synthesis was allocated with an initial degree of certainty based on the methodological design of included studies, i.e., low for observational (prospective epidemiological) studies and high for RCTs. Then, the degree of certainty was uprated or downrated based on 5 domains, namely risk of bias, inconsistency, imprecision, indirectness and publication bias. Two researchers (MG and AK) independently assessed each domain, any conflicts were discussed with and resolved by a third senior researcher (MK), and the certainty of evidence in each synthesis was rated as high, moderate, low, or very low. For the narrative evidence synthesis (without meta-analysis), the available guidance for rating the certainty of evidence in the absence of a single effect estimate was followed [18].

## 3. Results

### 3.1. Systematic Review Flowchart

**Figure 1:**
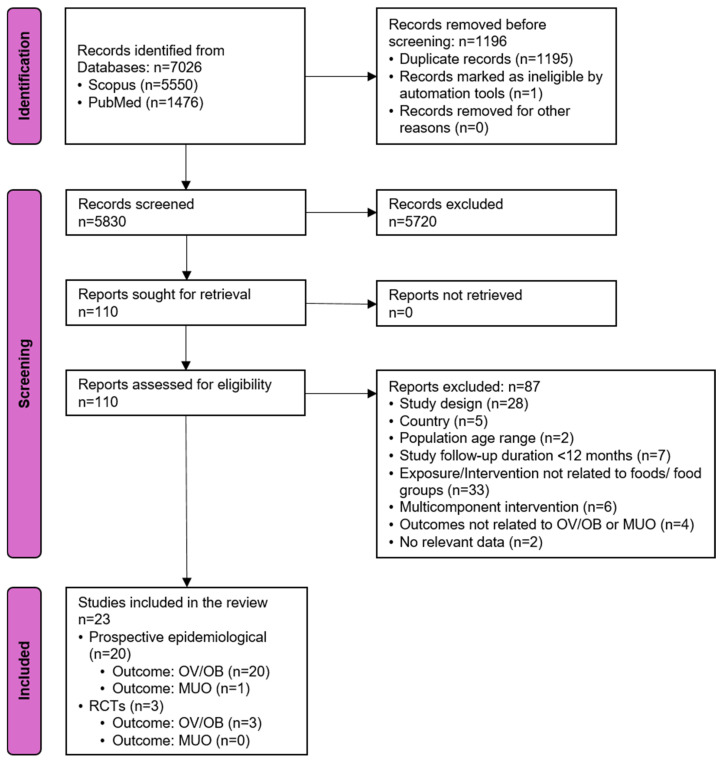
PRISMA flowchart. Abbreviations: MUO, metabolically unhealthy obesity; OV/OB, overweight/obesity; RCT, randomized controlled trial.

The study selection process is presented in Figure 1. The literature search yielded a total of 7026 studies (5550 from Scopus and 1476 from PubMed). After removing duplicates, 5830 studies were screened for eligibility based on titles/abstracts, of which 110 were potentially eligible and then reviewed in depth through full texts. Finally, a total of 23 studies examining the association between the consumption of foods/food groups and the risk of OV/OB and/or MUO in children 2–19 years of age were included in the systematic review, of which 20 were prospective epidemiological studies [19,20,21,22,23,24,25,26,27,28,29,30,31,32,33,34,35,36,37,38] and 3 were RCTs [39,40,41].

### 3.2. Study Characteristics

The characteristics of each included study are presented in detail in Supplementary Table S3 (prospective epidemiological studies) and Supplementary Table S4 (RCTs). Of the 23 studies, 10 were conducted in the USA [24,26,27,31,32,33,34,35,36,39], 5 in the UK [21,22,25,29,30], 3 in Denmark [19,28,37], 2 in Australia [23,38], 1 in the Netherlands [41], 1 in Germany [20], and 1 in Norway [40]. Study participants included both boys and girls, except for 1 study that included only girls [33]. The mean age of participants ranged from 2.1 years [23] to 13.4 years [34] in the prospective epidemiological studies and from 4.9 years [39] to 11.8 years [40] in the RCTs. The follow-up duration of prospective epidemiological studies ranged from 1 year [24] to 15 years [31], while that of RCTs ranged from 1.6 years [41] to 7 years [40]. Participants’ dietary habits, in terms of food/food group consumption, were evaluated through various tools, namely food frequency questionnaires (n = 2), 24 h dietary recalls (n = 3), dietary records (n = 9), questionnaires (n = 6), or a combination of the aforementioned methods (n = 2), or via monitoring of the consumption of the provided foods (n = 1). All 23 studies [20 prospective epidemiological [19,20,21,22,23,24,25,26,27,28,29,30,31,32,33,34,35,36,37,38,39,40,41] and 3 RCTs [39,40,41]] focused on childhood OV/OB, while 1 prospective epidemiological study also reported on MUO-related outcomes [32].

### 3.3. Risk of Bias

The assessment of risk of bias in the 20 eligible prospective epidemiological studies based on the ROBINS-E tool is presented in Table 1. Of the 20 studies, 12 (60%) were characterized as raising some concerns [19,22,24,25,27,28,30,31,32,35,36,37], while the remaining 8 (40%) were evaluated as having a low risk of bias [20,21,23,26,29,33,34,38]. The main contributors to risk of bias were confounding, arising from inadequate consideration of the obesogenic environment and sample characteristics (inclusion of participants with OV/OB at baseline) (domain 1); measurement of the exposure, due to self-reporting of dietary habits and diverse food serving sizes (domain 2); and selection of participants into the study (or into the analysis) due to non-representative study samples and missing data (domain 3). The assessment of risk of bias in the three eligible RCTs based on the RoB2 tool is presented in Table 2. Although concerns were raised in terms of deviations from intended interventions (domain 2) in two RCTs and due to missing data (domain 3) in one RCT, all three RCTs were evaluated as low risk of bias in total [39,40,41].

### 3.4. Qualitative Synthesis

#### 3.4.1. Risk of Childhood Overweight/Obesity

Of the 20 prospective epidemiological studies, 10 focused on SSBs [19,22,23,26,28,29,30,31,37,38], 8 on fruits and/or vegetables [19,20,26,31,33,34,36,38], 5 on artificially sweetened beverages (ASBs) [19,26,29,30,38], 7 on dairy products [19,21,26,31,32,35,38] and 3 on UPFs [24,25,27] (Supplementary Table S3). Accordingly, of the 3 RCTs, 1 focused on SSBs [41], 1 on SSBs and fast food [39], and 1 on fruits [40] (Supplementary Table S4). An overview of the main findings and the degree of certainty in evidence of the qualitative synthesis of studies sharing common exposures/interventions and outcomes is presented in Table 3.

##### Sugar-Sweetened Beverages

Among the 10 prospective epidemiological studies examining the association between SSB consumption and childhood OV/OB risk, 5 reported positive associations with various outcomes, namely body mass index (BMI) [22,29,30], BMI z-score [31,38], waist circumference (WC) [22], and total body fat mass [22] or % body fat [29,38], in some cases suggesting dose–response relationships [31,38]. Additionally, 1 study observed positive associations with BMI and WC that were attenuated after adjustment for total energy intake and other confounders [37], while another study reported a positive association with BMI z-score that was no longer significant after adjustment for total energy intake [19]. In contrast, 3 studies found no significant associations between SSB intake and various adiposity outcomes, including BMI [26,28], BMI z-score [23,28], WC [26,28], % body fat [26] and skinfold thickness [26,28]. Overall, the narrative synthesis was supportive of a detrimental association between SSB consumption and selected outcomes, i.e., BMI and % body fat, albeit the degree of certainty in evidence was low, while associations between SSB intake and other OV/OB-related outcomes were inconsistent/inconclusive (Table 3).

Among the 2 RCTs that focused on SSBs, 1 reported that replacing SSBs with sugar-free alternatives for 18 months resulted in a smaller increase in BMI z-score compared with continued consumption of SSBs, with stronger effects among children with higher baseline BMI [41]. In contrast, the other RCT showed that a 2-year behavioral intervention incorporating motivational interviewing and educational modules to reduce SSB and fast food intake did not significantly affect BMI z-score compared with usual care [39]. Overall, the qualitative synthesis was suggestive of an inconsistent/inconclusive effect of interventions targeting a decrease in SSB intake (either alone or combined with other foods) on childhood OV/OB-related outcomes with a low degree of certainty in evidence (Table 3).

##### Artificially Sweetened Beverages

Of the five prospective epidemiological studies evaluating the association between ASB consumption and childhood adiposity outcomes, one reported a positive association with increases in BMI and % body fat [29], whereas another reported an inverse association with change in BMI z-score and % body fat after adjustment for total energy intake [38]. In contrast, three studies reported no significant associations between ASB intake and various adiposity outcomes, including risk of OV/OB [30], BMI [26,30], BMI z-score [19], WC [26], % body fat [26], and sum of four skinfolds [26]. Overall, the narrative synthesis was suggestive of an inconclusive association between ASB intake and childhood OV/OB-related outcomes (BMI, BMI z-score and % body fat) with a very low to low degree of certainty in evidence (Table 3).

##### Dairy Products

Among the seven prospective epidemiological studies evaluating the association between consumption of dairy products and childhood adiposity outcomes, the following exposure categories were identified: (i) total dairy intake (n = 1), (ii) milk intake (quantity or frequency) (n = 5), and (iii) dairy consumption stratified by fat content (n = 3). For total dairy, the sole study revealed that a higher intake was associated with a smaller increase in BMI, although no association with the risk of excess body fat mass or OV/OB was evident [21]. Regarding milk intake (quantity or frequency), the five available studies reported no significant associations with adiposity outcomes, including risk of OV/OB [31], BMI [26], BMI z-score [19,31], WC [26], % body fat [31,37] and other adiposity measures [32]; in one of the abovementioned studies, lower milk intake in early childhood was associated with higher % body fat and greater skinfold thickness in adolescence [26]. Regarding dairy consumption stratified by fat content, the available three studies reported inverse associations between higher-fat dairy intake and adiposity outcomes. Specifically, full-fat dairy intake was associated with a smaller BMI increase and lower risk of excess body fat mass in one study [21]. Another study indicated that consumption of whole or 2% milk (vs. skim/low-fat milk) was associated with lower odds of OV/OB [32]. Similarly, 2%/whole milk consumption were inversely associated with BMI z-score and risk of OV/OB compared with lower-fat milk [35]. Overall, the narrative synthesis was suggestive of a null association between total milk intake and BMI z-score with a low degree of certainty in evidence, and a beneficial association between whole/higher-fat dairy products and the odds/risk of OV/OB with a very low degree of certainty in evidence (Table 3).

##### Fruits and Vegetables

Among the eight prospective epidemiological studies evaluating the association between fruit and/or vegetable consumption and childhood adiposity outcomes, evidence differed by exposure type, namely whole fruits/vegetables versus fruit/vegetable juice. The sole eligible study focusing on whole fruits and vegetables reported no significant association between their consumption and BMI z-score [20]. Regarding 100% fruit juice consumption, findings were mixed; one study reported a favorable association, with higher intake being associated with lower BMI [33], whereas one study reported a detrimental association, with higher intake being associated with increased BMI z-score and higher odds of OV [36]. In contrast, four studies reported no significant associations between 100% fruit juice intake and BMI percentile [34], BMI z-score [31], change in BMI [19,34], change in BMI z-score [38] or % body fat [38]. Additionally, one study examining combined fruit and vegetable juice intake as exposure reported inverse associations with WC and skinfold thickness, but no association with BMI or % body fat [26]. Overall, the narrative synthesis was suggestive of an inconsistent/inconclusive association between 100% fruit juice intake and childhood OV/OB-related outcomes (BMI and BMI z-score) with a very low degree of certainty in evidence (Table 3).

Only one RCT investigated the effects of a school-based intervention aimed at increasing fruit intake on childhood OV/OB risk [40]. In this study, the intervention group received free fruit for 9 months, while the control group received no intervention. At 4-year follow-up, the prevalence of OV was lower in the intervention group compared with controls (15% vs. 25%), corresponding to an unadjusted OR of 0.52 (95% CI: 0.28–0.97). However, this association was no longer statistically significant after adjustment for covariates and clustering, and no significant differences between groups were observed in BMI (Supplementary Table S4).

##### Ultra-Processed Foods

In the three prospective epidemiological studies examining UPF consumption in relation to childhood adiposity outcomes, UPF exposure was defined and operationalized using different approaches. In the studies of Carroll et al. [24] and Heerman et al. [27], UPF intake was defined according to the NOVA classification system as group 4 foods (industrial formulations with minimal intact food, such as chips, nuggets, and processed cereals). Carroll et al. [24] revealed no significant association between UPF intake and BMI, despite a relatively high UPF consumption. Heerman et al. [27] reported that a higher UPF intake (vs. lower intake) was associated with higher BMI z-scores at 36 months follow-up among children aged 3 and 4 years at baseline, but not among those aged 5 years or in overall analyses. In the study of Dong et al. [25], several foods typically considered as UPFs, such as desserts and sweets, SSBs, processed meats, coated fish, and potatoes cooked in oil, were evaluated as individual dietary components without the use of a formal system for UPF classification; the study revealed that sweets, processed meats, and SSBs were positively associated with 3-year excess weight gain.

#### 3.4.2. Risk of Childhood Metabolically Unhealthy Obesity

Only one prospective epidemiological study examined associations between foods/food groups and childhood MUO risk (Supplementary Table S3) [32]. In a subgroup of children with OV/OB, McGovern et al. [32] reported that higher-fat milk intake in early childhood was associated with more a favorable cardiometabolic profile, including lower HOMA-IR and higher adiponectin levels; however, similar associations were not observed among children with healthy weight, suggesting a differential pattern of associations by baseline body weight status. No RCTs evaluating dietary interventions in relation to MUO in children were identified.

### 3.5. Meta-Analysis

**Figure 2:**
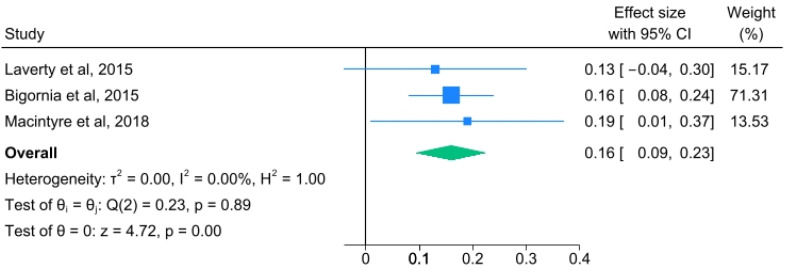
Forest plot for the association between SSB consumption and follow-up BMI. The vertical black line represents the null association. Abbreviations: BMI, body mass index; CI, confidence interval; SSB, sugar-sweetened beverage. Citations: Laverty et al., 2015 [29]; Bigornia et al., 2015 [22]; Macintyre et al., 2018 [30].

**Figure 3:**
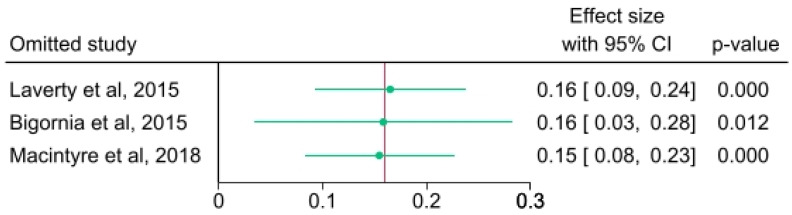
Leave-one-out sensitivity analysis for the association between SSB consumption and follow-up BMI. The vertical black line represents the null association. The vertical red line represents the pooled result of the main meta-analysis. Abbreviations: BMI, body mass index; CI, confidence interval; SSB, sugar-sweetened beverage. Citations: Laverty et al., 2015 [29]; Bigornia et al., 2015 [22]; Macintyre et al., 2018 [30].

**Figure 4:**
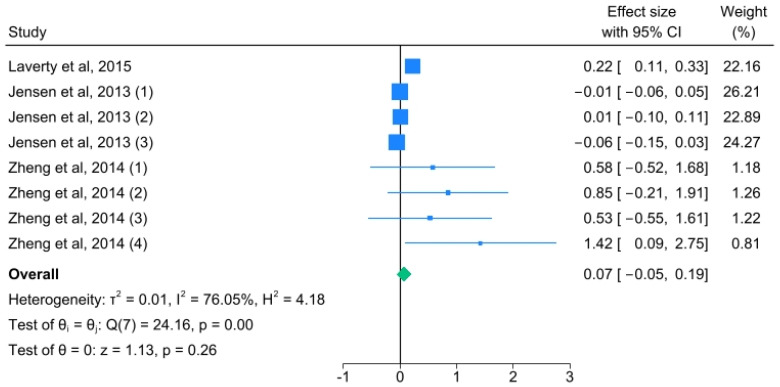
Forest plot for the association between SSB consumption and change in BMI. The vertical black line represents the null association. Jensen et al. (2013) (1) refers to the association between SSB intake at age 6 years and change in BMI between 6 and 9 years; Jensen et al. (2013) (2) refers to the association between SSB intake at age 9 years and change in BMI between 9 and 13 years; Jensen et al. (2013) (3) refers to the association between SSB intake at age 6 years and change in BMI between 6 and 13 years. Zheng et al. (2014) (1) refers to the association between SSB intake and change in BMI at the 6-year follow-up among children who consumed ≤1 serving/day; Zheng et al. (2014) (2) refers to the association between SSB intake and change in BMI at the 6-year follow-up among children who consumed >1 serving/day; Zheng et al. (2014) (3) refers to the association between SSB intake and change in BMI at the 12-year follow-up among children who consumed ≤1 serving/day; Zheng et al. (2014) (4) refers to the association between SSB intake and change in BMI at the 12-year follow-up among children who consumed >1 serving/day. Abbreviations: BMI, body mass index; CI, confidence interval; SSB, sugar-sweetened beverage. Citations: Laverty et al., 2015 [29]; Jensen et al., 2013 [28]; Zheng et al., 2014 [37].

**Figure 5:**
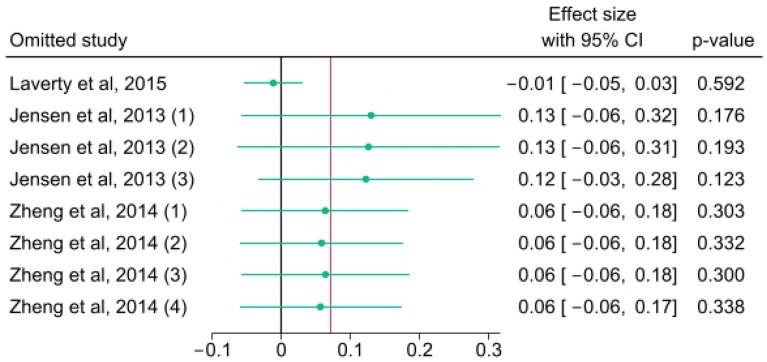
Leave-one-out sensitivity analysis for the association between SSB consumption and change in BMI. The vertical black line represents the null association. The vertical red line represents the pooled result of the main meta-analysis. Abbreviations: BMI, body mass index; CI, confidence interval; SSB, sugar-sweetened beverage. Citations: Laverty et al., 2015 [29]; Jensen et al., 2013 [28]; Zheng et al., 2014 [37].

Due to the limited number of included RCTs (n = 3), the potential of meta-analysis was only explored among prospective epidemiological studies (n = 20). A subgroup of three studies examining the association between SSB consumption and follow-up BMI [22,29,30] and another subgroup of three studies examining the association between SSB consumption and change in BMI [28,29,37] were meta-analyzed. In the first subgroup, two studies focused on the baseline frequency of SSB consumption (at least once a day vs. less than once a week or never) [29,30], while one focused on change in SSB consumption from baseline to follow-up (servings/day) [22]. Random-effects meta-analysis revealed a significant positive association between SSB consumption and follow-up BMI (pooled beta: 0.16 kg/m^2^, 95%CI: 0.09, 0.23) (Figure 2). Heterogeneity between studies was not present (I^2^ = 0.00%; Q = 0.23, *p* = 0.89), and results remained consistent in a leave-one-out sensitivity analysis (Figure 3); however certainty in evidence was low due to the observational nature of the studies. In the second subgroup, all three studies assessed SSB consumption at baseline but in different ways [baseline frequency (at least once a day vs. less than once a week or never) [29], 100 g (3.38 oz) [28], and ≤1 vs. >1 serve/day [37]]. Random-effects meta-analysis revealed a non-significant association between SSB intake and change in BMI (pooled beta: 0.07 kg/m^2^, 95%CI: −0.05, 0.19) (Figure 4). Heterogeneity between studies was high (I^2^ = 76.05%; Q = 24.16, *p* < 0.001), and results were consistent in a leave-one-out sensitivity analysis (Figure 5), while certainty in evidence was rated as very low due to inconsistency and imprecision. In both cases, due to the low number of meta-analyzed studies, publication bias could not be evaluated and meta-regressions could not be performed.

Meta-analysis of prospective epidemiological studies for the association between the consumption of the remaining foods/food groups and OV/OB-related outcomes was not feasible due to the limited number of studies sharing common outcomes and their substantial heterogeneity in exposures. Specifically, a total of five studies examined the odds of OV/OB as outcome [21,30,32,35,36], of which three focused on dairy products but with very heterogeneous exposures [one on total dairy, full-fat dairy, and low-fat dairy (higher vs. lower consumption) [21], two on milk type (whole/2% vs. 1%/skim) [32,35], one on milk frequency (times/day) [32]], one on 100% fruit juice [36], one on SSBs [30], and one on ASBs [30]. A total of 3 studies examined follow-up BMI as outcome [29,30,33], of which 2 focused on ASBs [29,30] and 1 on 100% fruit juice [33]. Change in BMI was evaluated as outcome in four studies [21,28,29,34], of which one focused on full-fat dairy [21], one on orange juice [34], one on sweet drinks (soft drinks, squash, fruit juice, chocolate milk and drinkable yoghurt) [28], one on soft drinks [28], two on SSBs [29,37], and one on ASBs [29]. A total of three studies examined follow-up BMI z-score as outcome [27,31,32], of which one focused on milk type (whole/2% vs. 1%/skim) [32], one on milk quantity (8 oz of additional daily intake) [31], one on 100% fruit juice [31], one on SSBs [31], and one on UPFs [27]. Change in BMI z-score was evaluated as outcome in five studies [19,20,36,37,38], of which two focused on milk [19,38], one on fruits [20], one on vegetables [20], two on 100% fruit juice [36,38], two on SSBs [19,38], and two on ASBs [19,38]. Another four prospective epidemiological studies did not share common outcomes and therefore could not be pooled or did not report the necessary effect estimates to be included in the meta-analysis [23,24,25,26].

## 4. Discussion

Research over the last years has provided novel insights into the obesogenic environment and specifically the link between the consumption of foods/food groups and the risk of OV/OB and MUO in children and adolescents [2,3,7]. However, the dearth of high-quality evidence from prospective epidemiological studies and RCTs necessitates an ongoing evidence synthesis in the field. The present systematic review and meta-analysis aimed to collect and synthesize long-term longitudinal evidence of the association between food/food group consumption and the risk of childhood OV/OB and MUO. According to the synthesis of the available data, consumption of SSBs emerged as the main food-related parameter associated with increased risk of childhood OV/OB, while a detrimental effect of UPFs and a beneficial effect of higher-fat dairy products on childhood OV/OB-related outcomes were also supported by limited evidence. Data for other foods/food groups, as well as studies evaluating the longitudinal association of foods/food groups with childhood MUO-related outcomes, were scarce or inconclusive.

In the present work, a positive association was evident between SSB consumption and childhood OV/OB-related outcomes based on the available long-term longitudinal evidence; this is supported by a narrative synthesis of 10 prospective epidemiological studies, of which 8 revealed positive associations, and 2 RCTs, of which 1 supports the beneficial effects of limiting SSBs, as well as a meta-analytic synthesis of a subgroup of 3 prospective epidemiological studies with BMI as an outcome. The observed detrimental effect aligns with previous literature reviews [42,43]. Their high sugar/energy content, as well as the reduced satiety and incomplete compensation reduction in energy intake at meals following the intake of liquid calories, can explain the detrimental effect of SSBs on children’s body weight status [44]. Regarding the link between ASB intake and childhood OV/OB, the literature remains inconclusive, as supported by a narrative synthesis of five prospective epidemiological studies (one with positive associations, one with inverse associations, three with non-significant findings). This inconsistency is also supported by previous works in the field; a systematic review and meta-analysis among 2–19 year-old children and adolescents indicated a non-significant association between the consumption of ASBs and BMI change in prospective epidemiological studies, while the pooled results from RCTs comparing ASBs with SSBs showed less BMI gain, especially among adolescents with OB [45]. More research in this area is advocated, especially as ASBs provide sweetness with little or no calories, taking into account the variety of non-nutritive sweeteners and their potential metabolic effects, as well as the methodological limitations of relevant research mostly pertinent to residual confounding and reverse causality [46].

Regarding UPFs, the present systematic review identified a positive association between their consumption and childhood OV/OB-related outcomes, supported by a narrative synthesis of three prospective epidemiological studies, of which two revealed positive associations. This finding aligns with previous systematic reviews of the literature, indicating that processed food consumption is among the primary dietary risk factors for childhood OV/OB [47,48]. While UPFs have been proposed as a key driver of adiposity and an important risk factor for non-communicable diseases in the modern nutritional epidemiology [49,50], several concerns have been raised regarding their link to health/disease outcomes [51]. These concerns are mostly pertinent to the heterogeneity in their definition and the methodological limitations of existing research that question whether it is the degree of processing per se or the adverse nutritional profile of UPFs that drive the risk for OV/OB and MUO, highlighting the need for more research with standardized methodologies for UPF assessment in the field [51].

Contrariwise, the present work identified an inverse relationship between full/higher-fat dairy products and childhood OV/OB-related outcomes, supported by a narrative synthesis of three prospective epidemiological studies of which two revealed beneficial associations. Dairy products, especially milk, are an important dietary source of protein and play an important role in optimal growth and development due to their high nutritional value [52]. Although total dairy consumption has been linked to a lower risk of childhood OV/OB [53,54,55], the type of dairy products consumed, in terms of their lipid content, in relation to body weight status remains a controversial topic. In accordance with the present findings, some previous works have revealed that consumption of whole/higher-fat dairy products, most importantly whole-fat milk, is beneficially associated with children’s body weight status [56,57,58,59]. However, this protective effect needs to be interpreted with caution, in light of other research data supporting the absence of a significant effect [60,61], as well as the fact that most of the available evidence is observational in nature and thus prone to residual confounding or reverse causality resulting from children with high risk of or prevalent OV/OB being advised on the beneficial effects of switching to low-fat dairy products. In this regard, national dietary guidelines for children aged 2 years and above in the UK, USA and Australia typically suggest the consumption of low-fat or skim against full-fat dairy products [62,63,64], on the basis of their reduced energy and saturated fat content [65]. The dearth of high-quality longitudinal studies and RCTs in the field highlight the need for further research to elucidate the potential beneficial association between consumption of whole/higher-fat dairy products and indicators of childhood OV/OB risk.

Regarding fruits and vegetables, the present narrative synthesis of eight prospective epidemiological studies and one RCT was inclusive on their association with childhood OV/OB-related outcomes. Fruits and vegetables are part of a healthy diet and are considered protective against excessive body weight and cardiometabolic pathology due to their low energy and high dietary fiber and micronutrient content. Previous systematic reviews and meta-analyses have produced mixed findings regarding their association with childhood OV/OB-related outcomes [66,67], a fact that has been attributed to heterogeneity in the methods used to assess and classify consumption and the significant variance in adjustment for other important potential confounders including energy intake. Regarding fruits juices, some concerns have been raised on the basis of their high content in free sugars and energy and lower content in dietary fibers compared to whole fruits, which could potentially lead to weight gain; nevertheless, although a relatively weak positive association between consumption of 100% fruit juice and adiposity indices has been observed among some cohorts of children and adolescents with OV/OB, the majority of the available evidence is suggestive of a non-significant link [68,69].

The present systematic review/meta-analysis has various strengths. In terms of its methodological framework, this includes a careful formulation of research questions based on the PECO/PICO models, a comprehensive and transparent reporting of the objectives, methodology and findings according to established guidelines (PRISMA and SWiM), the evaluation of studies’ risk of bias through valid tools endorsed by the Cochrane collaboration (ROBINS-E and ROB-2) and the assessment of the certainty in the available evidence using the GRADE tool. Furthermore, the applied evidence synthesis was based on data from studies with methodological designs of the highest quality (prospective epidemiological studies and RCTs); large sample sizes, sufficient to draw robust conclusions on the association between foods/food groups and OV/OB or MUO-related outcomes; standard tools for dietary assessment, including FFQs, dietary recalls, food diaries and interviews, ensuring a valid assessment of dietary exposures; and relatively low risk in terms of risk of bias.

However, some limitations of the present work require acknowledgment. The literature search was conducted only in two databases (Scopus and PubMed), a fact that may have led to missing relevant evidence, despite their comprehensive coverage of medical, nutritional epidemiology and public health literature; reference lists of included studies and previous systematic reviews in the filed were additionally searched to mitigate this risk. Furthermore, the present work focused only on studies conducted in countries of the Western world (Europe, Canada, the USA, and Oceania), which may have impacted the number of articles identified and the generalizability of findings in other settings; however, this was deemed necessary to limit heterogeneity, enhance comparability, and allow for a high-quality study synthesis. In addition, most of the included studies in the systematic review were observational in nature (prospective epidemiological) with a high degree of heterogeneity in terms of exposure definition, and thus are prone to several types of bias, residual confounding and reverse causation, limiting the strength, precision and causality of the findings of their synthesis. In terms of the quantitative synthesis, due to the limited number and the high heterogeneity of eligible studies, a meta-analysis was only feasible for a few studies exploring the longitudinal association between SSB consumption and BMI. The results of this meta-analysis should also be interpreted with caution, given the heterogeneity in exposure definition among studies which only allowed a generic estimation of the direction of the association (positive) and precluded its accurate quantification, as well as the inability to explore publication bias and perform meta-regressions due to the limited number of included studies. Last but not least, the present work was unable to produce robust conclusions regarding the association between foods/food groups and childhood MUO risk, due to the paucity of relevant research, and highlights this topic as an understudied field.

## 5. Conclusions

The present systematic review and meta-analysis sought to shed light into the association between the consumption of foods/food groups and the risk of childhood OV/OB and MUO. The available evidence from studies with a longitudinal design support that SSB consumption is positively associated with indicators of OV/OB risk, while limited data also suggest a potential detrimental effect of UPFs and a potential beneficial effect of higher-fat dairy products. However, the available studies were few and mostly of observational nature, thereby limiting the strength and quality of evidence. Research data for other foods/food groups in relation to OV/OB, as well as for the association between foods/food groups and MUO, remain limited and/or inconclusive. Given that evidence of the association between consumption of foods/food groups and health outcomes can influence clinical practice and public health strategies, additional research is needed to inform dietary strategies for the prevention of childhood OV/OB and its cardiometabolic burden in childhood and adolescence.

## Figures and Tables

**Table 1:** Risk of bias assessment in prospective epidemiological studies.

Study (Author, Year)	D1	D2	D3	D4	D5	D6	D7	Overall
Bayer et al., 2014 [20]	+	−	+	+	+	+	+	+
Bigornia et al., 2014 [21]	+	+	+	+	−	+	+	+
Bigornia et al., 2015 [22]	−	+	+	+	−	+	+	−
Byrne et al., 2018 [23]	+	+	+	+	+	+	+	+
Carroll et al., 2024 [24]	−	−	−	+	+	+	+	−
Dong et al., 2015 [25]	+	−	−	+	+	+	+	−
Hasnain et al., 2014 [26]	+	+	+	+	+	+	+	+
Heerman et al., 2023 [27]	−	−	+	+	+	−	+	−
Jensen et al., 2013 [28]	−	−	+	+	−	+	+	−
Laverty et al., 2015 [29]	−	+	+	+	+	+	+	+
Macintyre et al., 2018 [30]	−	−	+	+	+	+	+	−
Marshall et al., 2019 [31]	−	−	+	+	+	+	+	−
McGovern et al., 2022 [32]	−	−	−	+	+	+	+	−
Moore et al., 2023 [33]	+	−	+	+	+	+	+	+
Sakaki et al., 2021 [34]	+	−	+	+	+	+	+	+
Scharf et al., 2013 [35]	−	−	+	+	+	−	+	−
Shefferly et al., 2016 [36]	−	−	+	+	+	+	+	−
Zheng et al., 2014 [37]	+	−	−	+	+	−	+	−
Zheng et al., 2015 [38]	+	−	−	+	+	+	+	−
Zheng et al., 2015 [19]	+	+	+	+	+	+	+	+

D1: bias due to confounding, D2: bias arising from measurement of the exposure, D3: bias in selection of participants into the study (or into the analysis), D4: bias due to post-exposure interventions, D5: bias due to missing data, D6: bias arising from measurement of the outcome, D7: bias in selection of the reported result. Symbols: +, low risk of bias; −, some concerns. Abbreviations: D, domain.

**Table 2:** Risk of bias assessment in randomized controlled trials.

Study (Author, Year)	D1	D2	D3	D4	D5	Overall
Bere et al., 2014 [40]	+	+	−	+	+	+
Katan et al., 2016 [41]	+	−	+	+	+	+
Rifas-Shiman et al., 2017 [39]	+	−	+	+	+	+

D1: bias arising from the randomization process, D2: bias due to deviations from intended interventions, D3: bias due to missing outcome data, D4: bias in the measurement of the outcome, D5: bias in the selection of the reported result. Symbols: +, low risk of bias; −, some concerns. Abbreviations: D, domain.

**Table 3:** Summary of findings from qualitative evidence synthesis.

Food/Food Group	Outcome	Study Design	Number of Studies (Participants)	Direction of Combined Effect	Certainty in Evidence *	References
SSB	BMI (FU and Δ)	PES	6 (19,358)	Positive association	⊕⊕◯◯	[21,25,27,28,29,36]
SSB	BMI z-score	RCT	2 (888)	Inconclusive effect (beneficial/null)	⊕⊕◯◯ (downgraded due to inconsistency)	[38,40]
SSB	BMI z-score (FU and Δ)	PES	5 (1902)	Inconclusive association (positive/null)	⊕◯◯◯ (downgraded due to inconsistency and imprecision)	[18,22,27,30,37]
SSB	WC (FU and Δ)	PES	4 (3202)	Inconclusive association (positive/null)	⊕⊕◯◯	[21,25,27,36]
SSB	BF% (FU and Δ)	PES	3 (13,426)	Positive association	⊕⊕◯◯	[25,28,37]
ASB	BMI (FU)	PES	3 (16,254)	Inconclusive association (positive/null)	⊕⊕◯◯	[25,28,29]
ASB	BMI z-score (Δ)	PES	2 (510)	Inconclusive association (positive/null)	⊕◯◯◯ (downgraded due to inconsistency and imprecision)	[18,37]
ASB	% body fat (FU and Δ)	PES	3 (13,426)	Inconclusive association (positive/negative/null)	⊕◯◯◯ (downgraded due to inconsistency)	[25,28,37]
Milk	BMI z-score (FU and Δ)	PES	4 (1929)	No association	⊕⊕◯◯	[18,30,31,37]
Full-fat milk	OV/OB (odds and risk)	PES	2 (9096)	Inverse association	⊕◯◯◯ (downgraded due to risk of bias)	[31,34]
100% fruit juice	BMI (FU and Δ)	PES	3 (9818)	Inconclusive association (negative/null)	⊕◯◯◯ (downgraded due to inconsistency)	[18,32,33]
100% fruit juice	BMI z-score (FU and Δ)	PES	3 (9731)	Inconclusive association (positive/null)	⊕◯◯◯ (downgraded due to inconsistency and imprecision)	[30,35,37]

* Symbols used to describe certainty in the evidence: ⊕⊕◯◯, low certainty; ⊕◯◯◯ very low certainty. Abbreviations: ASB: artificially sweetened beverage; BF%: body fat percentage; BMI: body mass index; FU: follow-up; OV/OB: overweight/obesity; PES: prospective epidemiological study; RCT: randomized controlled trial; SSB: sugar-sweetened beverage; WC: waist circumference, Δ: change.

## Data Availability

All data generated or analyzed during this study are included in this article (and its Supplementary Materials Files). Further enquiries can be directed to the corresponding author.

## Supplementary Materials

The following supporting information can be downloaded at: https://www.mdpi.com/article/10.3390/life16060934/s1, Table S1: PRISMA checklist; Table S2: Search query; Table S3: Overview of prospective epidemiological studies examining the association between foods/food groups and childhood OV/OB risk; Table S4: Overview of RCTs examining the effect of interventions targeting foods/food groups on the risk of childhood OV/OB.

## Abbreviations

Artificially Sweetened Beverage (ASB); Body Mass Index (BMI); Metabolically Unhealthy Obesity (MUO); Obesity (OB); Overweight (OV); Population-Exposure-Comparator-Outcome (PECO); Population-Intervention-Comparator-Outcome (PICO); Preferred Reporting Items for Systematic reviews and Meta-analyses (PRISMA); Randomized Controlled Trial (RCT); Risk of Bias in Non-randomized Studies of Exposures (ROBINS-E); Sugar-Sweetened Beverage (SSB); Synthesis Without Meta-analysis (SWiM); Ultra-Processed Food (UPF); Waist Circumference (WC).
