# Supplementary material for: The Association Between Consumption of Foods/Food Groups and the Risk of Overweight/Obesity and Metabolically Unhealthy Obesity in Children and Adolescents: A Systematic Review and Meta-Analysis
Source: Life (Basel). 2026 Jun 1;16(6):934. doi: 10.3390/life16060934 (PMC13302376; doi:10.3390/life16060934)
Supplement: Supplementary file 1 [file life-16-00934-s001.zip › life-4183286-supplementary.pdf]

# SUPPLEMENTARY LIST

# The association between consumption of foods/ food groups and the risk of overweight/obesity and metabolically unhealthy obesity in children and adolescents: A systematic review and meta-analysis

**Fidelia Bature[1], Michael Georgoulis[2], Athanasia Kyrkili[2], Meropi D. Kontogianni[2], Zoi-Eleni Koti[3], Chara Kapsala[3], Iliana Korma[3] Yannis Pappas[1*]**

[1] Institute for Health Research, Putteridge Bury Campus, University of Bedfordshire, Luton, LU28LE.

[2] Department of Nutrition and Dietetics, School of Health Sciences and Education, Harokopio University of Athens, 17676 Athens, Greece.

[3] UKEMED GLOBAL LTD. 121, Prodromou Street Offices 713-715, 2064 Nicosia, Cyprus.

* Corresponding author: Professor Yannis Pappas, Institute for Health Research, Putteridge Bury Campus, University of Bedfordshire, Luton, LU2 8LE.

## Supplementary Table S1. PRISMA checklist.

| Section and Topic | Item # | Checklist item | Location where item is reported* |
|---|---|---|---|
| **TITLE** | | | |
| Title | 1 | Identify the report as a systematic review. | Page 1 |
| **ABSTRACT** | | | |
| Abstract | 2 | See the PRISMA 2020 for Abstracts checklist. | Pages 2-3 |
| **INTRODUCTION** | | | |
| Rationale | 3 | Describe the rationale for the review in the context of existing knowledge. | Pages 4-5 |
| Objectives | 4 | Provide an explicit statement of the objective(s) or question(s) the review addresses. | Page 5 |
| **METHODS** | | | |
| Eligibility criteria | 5 | Specify the inclusion and exclusion criteria for the review and how studies were grouped for the syntheses. | Pages 6-7 |
| Information sources | 6 | Specify all databases, registers, websites, organisations, reference lists and other sources searched or consulted to identify studies. Specify the date when each source was last searched or consulted. | Pages 7-8 |
| Search strategy | 7 | Present the full search strategies for all databases, registers and websites, including any filters and limits used. | Pages 7-8, Supplementary Table S2 |
| Selection process | 8 | Specify the methods used to decide whether a study met the inclusion criteria of the review, including how many reviewers screened each record and each report retrieved, whether they worked independently, and if applicable, details of automation tools used in the process. | Pages 8-9, Data extraction and synthesis |
| Data collection process | 9 | Specify the methods used to collect data from reports, including how many reviewers collected data from each report, whether they worked independently, any processes for obtaining or confirming data from study investigators, and if applicable, details of automation tools used in the process. | Pages 8-9, Data extraction and synthesis; Pages 9-10, Certainty of evidence |
| Data items | 10a | List and define all outcomes for which data were sought. Specify whether all results that were compatible with each outcome domain in each study were sought (e.g. for all measures, time points, analyses), and if not, the methods used to decide which results to collect. | Pages 8-9, Supplementary Tables S3 and S4 |
| | 10b | List and define all other variables for which data were sought (e.g. participant and intervention characteristics, funding sources). Describe any assumptions made about any missing or unclear information. | Pages 8-9, Supplementary Tables S3 and S4 |
| Study risk of bias assessment | 11 | Specify the methods used to assess risk of bias in the included studies, including details of the tool(s) used, how many reviewers assessed each study and whether they worked independently, and if applicable, details of automation tools used in the process. | Page 8, Tables 1 and 2 |
| Effect measures | 12 | Specify for each outcome the effect measure(s) (e.g. risk ratio, mean difference) used in the synthesis or presentation of results. | Pages 8-9 |
| Synthesis methods | 13a | Describe the processes used to decide which studies were eligible for each synthesis (e.g. tabulating the study intervention characteristics and comparing against the planned groups for each synthesis (item #5)). | Pages 8-9, Supplementary Tables S3 and S4 |
| | 13b | Describe any methods required to prepare the data for presentation or synthesis, such as handling of missing summary statistics, or data conversions. | Pages 8-9 |

| Section and Topic | Item # | Checklist item | Location where item is reported* |
|---|---|---|---|
| | 13c | Describe any methods used to tabulate or visually display results of individual studies and syntheses. | Pages 8-10 |
| | 13d | Describe any methods used to synthesize results and provide a rationale for the choice(s). If meta-analysis was performed, describe the model(s), method(s) to identify the presence and extent of statistical heterogeneity, and software package(s) used. | Pages 8-10 |
| | 13e | Describe any methods used to explore possible causes of heterogeneity among study results (e.g. subgroup analysis, meta-regression). | Pages 8-10 |
| | 13f | Describe any sensitivity analyses conducted to assess robustness of the synthesized results. | Pages 8-10 |
| Reporting bias assessment | 14 | Describe any methods used to assess risk of bias due to missing results in a synthesis (arising from reporting biases). | Pages 8-10 |
| Certainty assessment | 15 | Describe any methods used to assess certainty (or confidence) in the body of evidence for an outcome. | Pages 8-10 |
| **RESULTS** | | | |
| Study selection | 16a | Describe the results of the search and selection process, from the number of records identified in the search to the number of studies included in the review, ideally using a flow diagram. | Pages 10-11, Figure 1 |
| | 16b | Cite studies that might appear to meet the inclusion criteria, but which were excluded, and explain why they were excluded. | N/A |
| Study characteristics | 17 | Cite each included study and present its characteristics. | Supplementary Tables S3 and S4 |
| Risk of bias in studies | 18 | Present assessments of risk of bias for each included study. | Page 11, Tables 1 and 2 |
| Results of individual studies | 19 | For all outcomes, present, for each study: (a) summary statistics for each group (where appropriate) and (b) an effect estimate and its precision (e.g. confidence/credible interval), ideally using structured tables or plots. | Supplementary Tables S3 and S4 |
| Results of syntheses | 20a | For each synthesis, briefly summarise the characteristics and risk of bias among contributing studies. | Pages 11-16, Table 3 |
| | 20b | Present results of all statistical syntheses conducted. If meta-analysis was done, present for each the summary estimate and its precision (e.g. confidence/credible interval) and measures of statistical heterogeneity. If comparing groups, describe the direction of the effect. | Pages 11-16, Table 3 |
| | 20c | Present results of all investigations of possible causes of heterogeneity among study results. | Pages 11-16, Table 3 |
| | 20d | Present results of all sensitivity analyses conducted to assess the robustness of the synthesized results. | N/A |
| Reporting biases | 21 | Present assessments of risk of bias due to missing results (arising from reporting biases) for each synthesis assessed. | Pages 11-16, Table 3 |
| Certainty of evidence | 22 | Present assessments of certainty (or confidence) in the body of evidence for each outcome assessed. | Pages 11-16, Table 3 |
| **DISCUSSION** | | | |
| Discussion | 23a | Provide a general interpretation of the results in the context of other evidence. | Pages 17-21 |

| Section and Topic | Item # | Checklist item | Location where item is reported* |
|---|---|---|---|
| | 23b | Discuss any limitations of the evidence included in the review. | Pages 21-22 |
| | 23c | Discuss any limitations of the review processes used. | Pages 21-22 |
| | 23d | Discuss implications of the results for practice, policy, and future research. | Page 22 |
| **OTHER INFORMATION** | | | |
| Registration and protocol | 24a | Provide registration information for the review, including register name and registration number, or state that the review was not registered. | Page 6 |
| | 24b | Indicate where the review protocol can be accessed, or state that a protocol was not prepared. | Page 6 |
| | 24c | Describe and explain any amendments to information provided at registration or in the protocol. | N/A |
| Support | 25 | Describe sources of financial or non-financial support for the review, and the role of the funders or sponsors in the review. | Page 33 |
| Competing interests | 26 | Declare any competing interests of review authors. | Page 33 |
| Availability of data, code and other materials | 27 | Report which of the following are publicly available and where they can be found: template data collection forms; data extracted from included studies; data used for all analyses; analytic code; any other materials used in the review. | Page 33 |

* Page numbers refer to the submitted version of the manuscript.

**Supplementary Table S2. Search query#.**

| | |
|---|---|
| **Population** | (“child*” OR “adolescen*” OR “teen” OR “teenage*” OR “preteen*” OR “youth” OR “paediatric” OR “pediatric” OR “juvenile*” OR “youngster*” OR “girl” OR “girls” OR “boy” OR “boys” OR “kid” OR “kids” OR “young person*” OR “young people” OR “schoolchild*” OR “school age” OR “schoolage*” OR “student*” OR “pupil*”) |
| **Exposures/ interventions** | **AND** (“food*” OR “fruit*” OR “vegetable*” OR “legume*” OR “pulse*” OR “bean*” OR “nut” OR “nuts” OR “cereal*” OR “grain*” OR “dairy” OR “milk” OR “meat*” OR “poultry” OR “fish” OR “seafood” OR “beverage*” OR “drink*” OR “coffee” OR “tea” OR “sweet*” OR “ultra processed”) **AND** (“consumption” OR “intake”) |
| **Outcomes** | **AND** (“weight” OR “body mass index” OR “BMI” OR “overweight” OR “obesity” OR “adipos*” OR “body fat” OR “fatness” OR “skinfold thickness” OR “waist circumference” OR “fat mass” OR “fat free mass” OR “muscle mass” OR “neck circumference” OR “waist-to-height” OR “body composition” OR “*hypertension” OR “blood pressure” OR “dyslipidaemia” OR “dyslipidemia” OR “hyperlipidaemia” OR “hyperlipidemia” OR “hypercholesterolaemia” OR “hypercholesterolemia” OR “blood lipid*” OR “cholesterol” OR “triglyceride*” OR “blood glucose” OR “glycated haemoglobin” OR “glycated hemoglobin” OR “glycaemic control” OR “glycemic control” OR “HOMA” OR “HOMA-IR” OR “insulin resistance” OR “prediabetes” OR “metabolic syndrome” OR “inflammation” OR “liver enzymes” OR “hepatic steatosis” OR “non-alcoholic fatty liver disease” OR “NAFLD” OR “polycystic ovary syndrome” OR “PCOS” OR “obstructive sleep apnoea” OR “obstructive sleep apnea” OR “OSA” OR “uric acid” OR “hyperuricaemia” OR “hyperuricemia”) |
| **Study design** | **AND** (“prospective*” OR “longitudinal*” OR “randomized clinical trial” OR “randomised clinical trial” OR “randomized controlled trial” OR “randomised controlled trial” OR “incidence” OR “relative risk” OR “risk ratio” OR “RR” OR “hazard ratio” OR “HR”) |

**#The following filters were applied:**

- Scopus: search within (article title, abstract, keywords), date range (from 2013 to present), language (English), territory (all European countries, USA, Canada, Australia, New Zealand) and document type (article).
- PubMed: search within (title/abstract), species (humans), article language (English), age (preschool child: 2-5 years, child: 6-12 years, adolescent: 13-18 years) and publication date (custom range from 01/01/2013 to 30/06/2024).

**Supplementary Table S3. Overview of prospective epidemiological studies examining the association between foods/ food groups and childhood OV/OB risk.**

| Study | | Country | Setting | Population | | | FU | Exposures | Outcomes | Statistical analysis | Results |
|---|---|---|---|---|---|---|---|---|---|---|---|
| Author, year | Title (acronym) | | | N | Age [a] | Sex [b] | | | | | |
| Bayer et al, 2014 | Gesundheits-Monitoring-Einheiten, health-monitoring units<br>(GME) | Germany | School | 1,252<br>2,409 at BL<br>1,252 analysed after excluding lost to FU (48%) | 6.0±0.45 y at BL | M: 616 (49.2%)<br>F: 636 (50.8%) | 4 y | Fruit & vegetable consumption<br>Amount: >2 portions/day of fruits and vegetables defined as high<br>Dietary assessment: questionnaire. | Δ BMI z-score | Statistical model: generalized estimating equation models with ΔBMI z-score (Spearman's correlation rho)<br>Covariates: physical activity | No statistically significant associations were observed between longitudinal changes in fruit and vegetable consumption and BMI z-score; fruit intake showed no effect (p=0.236), increasing vegetable intake showed a non-significant tendency toward higher BMI gain compared with decreasing or consistently low intake (p= 0.230 and p=0.137). |
| Bigornia et al, 2014 | Avon Longitudinal Study of Parents and Children (ALSPAC) | England | Clinics & community (mothers) | 2,455<br>5,102 at BL with anthropometric and DXA measures<br>2,455 (48.1%) with dietary records analysed. | 10.6±0.2 y at BL;<br>13.8±0.2 y at FU | M: 1301 (53%)<br>F:1154 (47%) | 3 y<br>(3.2 ± 0.2 y) | Total dairy intake: white milk, flavoured milk, cheese, yogurt, ice cream made with dairy, and other dairy desserts.<br>Amount: 563 6 155 (378–868) g/d vs 88 6 54 (0–177) g/d.<br>Reduced-fat dairy intake: semi-skimmed (1.7%) or skimmed milk and any reduced-fat cheese | FU OV (International Obesity Taskforce age- and sex-specific weight categories),<br>Δ BMI,<br>Excess TBFM (defined as the top quintile for sex-specific and age- and height-adjusted total body fat mass) | Statistical model: multivariable logistic regression [OR (95% Cl) & mean (95% CI) changes]<br>Covariates: sex, age, height, adiposity, FU dairy intake, BL BMI for TBFM, maternal education, maternal OV, physical activity, pubertal stage, FU dieting, BL intakes of cereal, total fat, total protein, fiber, 100% fruit juice, fruit & vegetables, sugar-sweetened beverages), FU dietary reporting errors, and BL energy intake. | Children with the highest intakes of total dairy tended (P < 0.1) to have smaller gains in BMI from ages 10 to 13 y compared with those with the lowest intakes of dairy, with no increased risk of excess TBFM (p = 0.18) or OV (p = 0.07) at age 13 y.<br>The highest versus lowest quartile of total dairy intake was not associated with an increased risk of excess TBFM (p = 0.18) or OV (p = 0.07) at age 13 y. High versus low intake of full-fat dairy was associated with a reduced risk of excess TBFM (OR: 0.59; 95% CI: |

| | | | | | | | | | | | |
|---|---|---|---|---|---|---|---|---|---|---|---|
| | | | | | | | | (including cottage cheese) or yogurt product.<br>Amount: 439 6 154 (265–769) g/d vs 9 6 20 (0–50) g/d.<br>Full-fat dairy intake: products made with whole milk.<br>Amount: 348 6 176 (154–709) g/d vs 9 6 10 (0–26) g/d.<br>Dietary assessment: 3-day dairy records. | | | 0.37, 0.94; p = 0.02) and with smaller gains in BMI (OR: 2.5; 95% CI: 2.2, 2.7; p = 0.009) at age 13 y, but was not associated with risk of OV (p = 0.19).<br>No association was observed between reduced-fat dairy intake at age 10 y and excess adiposity at age 13 y (p > 0.05). |
| Bigornia et al, 2015 | Avon Longitudinal Study of Parents and Children (ALSPAC) | England | Clinics & community (mothers) | 2,455<br>5,102 at BL<br>2,455 analysed after excluding 2,647 (52%) of those with missing data | 10.6 ±0.2 y at BL;<br>13.8±0.2 y at FU | M: 1154 (47%)<br>F: 1301 (53%) | 3 y | SSB intake: full-sugar fruit squashes, cordials and fizzy drinks (i.e. soda) with added sugar, reduced-sugar or artificially sweetened versions as diet beverages.<br>Amount: ΔSSB =SSB (servings/d) at age 13 y – SSB (servings/d) at age 10 y. | FU BMI,<br>FU WC,<br>FU total body fat mass | Statistical model: Multilinear linear regression models (standardized β)<br>Covariates: age, sex, SSB intake, adiposity, FU physical activity, FU pubertal stage, maternal OV/OB, maternal education, FU dieting, changes in intakes of fruit juice, fruits & vegetables & total fat, dietary reporting errors, FU plausible dietary reporters | Increased SSB consumption from ages 10 to 13 years was associated with higher WC (standardized β=0,097, P<0,001), BMI (β=0,074, P<0,001), and total body fat mass (β=0,065, P=0,003) at 13 y.<br>The association between change in SSB and WC is weak, but statistically significant after accounting for BMI (β=0,042, P=0,02) and total body fat mass (β=0,048, P=0,01). |

| | | | | | | | | | | | |
|---|---|---|---|---|---|---|---|---|---|---|---|
| | | | | | | | | Dietary assessment: 3-day dairy records. | | | |
| Byrne et al, 2018 | NOURISH | Australia | Community | 403<br>(515 at 2 y, 426 at 3.7 y, 405 at 5 y)<br>403 analysed after removing 2 (0.49%) missing data | 2.1±0.7 y | M: 196 (48%)<br>F: 209 (52%) | 3 y | Sweet beverages: flavoured milks, 100% juice, dilute juice, fruit drink/cordial & soft drink<br>Amount: g/kg body weight<br>Dietary assessment: 3-pass-24h dietary recall | FU BMI-z-score | Statistical model: structural equation modelling<br>Covariates: NR | No relationship between intake of sweet beverages and BMI-z-score at any age. |
| Carroll et al, 2024 | NA | USA | Community | 560<br>624 at BL<br>560 (90%) with completed BL study analysed | 3.8±1.0 y | M:247 (44.1%)<br>F:313 (59.9%) | 1 y | UPF: NOVA Group 4, industrial formulations with minimal intact food (e.g., chips, nuggets, cereals)<br>Amount: Not specified (expressed as total intake/percentage of energy intake)<br>Dietary assessment: 2 3-day food records | Δ BMI | Statistical model: linear regression model (β)<br>Covariates: age, hours of television watching, parent soda/fast-food consumption frequency, parent education level, parent-reported race/ethnicity | No association between UPF intake and BMI. |
| Dong et al, 2015 | Avon Longitudinal Study of Parents and Children | UK | Community | 4,646<br>15,444 at BL<br>4,646 (30%) with completed data analysed | 11.5±0.1 y | M:2323 (50%)<br>F: 2323 (50%) | 3 y | UPF: desserts and sweets (including sweet biscuits, cookies; pudding; cake; ice cream; and | Weight gain<br>(a 0.01 increase in BMI z-score corresponded to 50 grams of | Statistical model: multivariable linear regression models<br>Covariates: age, sex, physical activity, puberty | Foods with the largest positive associations with three-year excess weight gain were fat spread (butter or margarine), coated (breaded or battered) |

| | | | | | | | | | | | |
|---|---|---|---|---|---|---|---|---|---|---|---|
| | | | | | | | | confectionery, or candy), refined grains, vegetables, fruit, potatoes, & poultry.<br>Amount: g/day<br>Dietary assessment: 3-day food diary | excess weight gain) | status (Tanner stage), maternal highest education | poultry, potatoes cooked in oil (French fries, roasted potatoes, and potato chips), coated fish, processed meats, other meats, desserts and sweets, milk, and sugar-sweetened beverages ($p<0.05$). |
| Hasnain et al, 2014 | Framingham Children's Study (FCS) | USA | Community | 98<br>106 at BL<br>103 had some dietary records.<br>98 (92.4%) analysed with complete data | 3-5 y at BL;<br>15-17 y at FU | M:47 (48%)<br>F:51 (52%) | 12 y | Milk: both plain and flavoured varieties plus small amounts of soymilk & rice beverages.<br>Fruit and vegetable juices: unsweetened fruit juice & small intakes of sweetened fruit & vegetable juices<br>SSBs: sweetened carbonated beverages, sweetened noncarbonated beverages, sweetened tea or coffee, & part-juice beverages.<br>Unsweetened (or artificially sweetened) beverages: diet/artificially sweetened carbonated and | FU BMI,<br>FU WC,<br>FU S4SF,<br>FU % body fat | Statistical model: longitudinal mixed models.<br>Covariates: sex, BL body fat, age at time of anthropometry, physical activity, percent of energy from fat, total energy intake, television/video viewing time, Tanner stage, maternal age, education level and BMI. | Children with the lowest milk intakes in early childhood had 7.4% more body fat (30.0% body fat in tertile 1 vs. 22.6% in tertile 3; $p=0.0095$) and lower S4SF ($p=0.0465$) in later adolescence than those with higher intakes. No association between milk intake and WC and BMI.<br>Children with the highest tertile of fruit and vegetable juice intake during childhood had an 8.0 cm smaller WC ($p=0.0328$) and lower S4SF ($p=0.0383$) at 15–17 years of age, compared with those in the lowest tertile. No association between fruit and vegetable juice intake and % body fat and BMI.<br>No association between SSBs and % body fat ($p=0.9296$) or other outcomes ($p>0.05$). |

| | | | | | | | | | | |
|---|---|---|---|---|---|---|---|---|---|---|
| | | | | | | | | noncarbonated beverages, as well as unsweetened & artificially sweetened tea or coffee.<br>Amount: oz/ day<br>Dietary assessment: 3 days dietary records | | | |
| Heerman et al, 2023 | Growing Right Onto Wellness (GROW) trial | USA | Clinics & community | 595<br>610 at BL<br>595 (97.5%) analysed that had complete data | 4.3 (3.6–5.0) y | M:284 (47.7%)<br>F: 311 (52.3%) | 3 y | UPF: NOVA Group 4<br>Amount: 1300 kcals/day vs 300 kcals/day<br>Dietary assessment: 3 24-h diet recall | FU BMI z-score | Statistical model: longitudinal mixed effects linear regression analysis stratified by baseline age group (3-, 4-, and 5-year-olds)<br>Covariates: BL UPF intake (kcal), total energy intake, age, sex, mean daily percent of time spent in moderate-vigorous physical activity, parent ethnicity, household food security, WIC/SNAP participation, RCT group, and timepoint × age interactions. | Compared with low UPF consumption, high UPF intake was associated with a 1.2 higher BMI z-score at 36 months for 3-year-olds (95% CI = 0.5, 1.9; p < 0.001) and a 0.6 higher BMI-z-score for 4-year-olds (95% CI = 0.2, 1.0; p= 0.007).<br>The difference was not statistically significant for 5-year-olds (-0.1; 95% CI -0.6, 0.4; p=0.7) or overall (0.4; 95% CI -0.02, 0.7; p=0.07). |
| Jensen et al, 2013 | Copenhagen School Child Intervention Study<br>(CoSCIS) | Denmark | School setting | 366<br>1,024 invited at 6 years<br>366 (36%) analysed with complete data | 6.7±0.3 y at BL;<br>9.5±0.4 y & 13.3±0.3 y at FU | M: 179 (48.9%)<br>F: 187 (51.1%) | 3 & 7 y | Sweet drink: soft drink, squash, fruit juice, chocolate milk and drinkable yogurt & a subgroup SSBs including intake of soft drink and squash, only. | Δ BMI,<br>Δ log S4SF | Statistical model: multilinear regression models<br>Covariates: BL BMI or log S4SF and school cluster, sex, intervention group, pubertal status, socio-economic status, total energy intake, physical | No associations were observed between sweet drink intake at age 6 y and change in BMI or log S4SF from age 6 to 9 y or 6 to 13 y.<br>No associations were observed between changes in sweet drink intake from age 6 to 9 y and subsequent |

| | | | | | | | | | | | |
|---|---|---|---|---|---|---|---|---|---|---|---|
| | | | | | | | | Amount: 100ml=100g<br>Dietary assessment: 7 days food record | | activity, maternal BMI, & beverage intake. | change in BMI or logS4SF from age 9 to 13 y.<br>A weak direct association was observed between sweet drink intake at age 9 y and change in logS4SF from age 9 to 13 y (per 100 g ~ 3.38 fl oz) (b: 0.014; 95% CI: -0.001, 0.029; p=0.06), while no association was observed for BMI. |
| Laverty et al, 2015 | Millennium Cohort Study (MCS) | UK | General population | 13,170<br>13,287 at BL<br>13,170 analysed after excluding 117 (0.8%) with missing data | 10.9±0.9 y | M:6651 (50.5%)<br>F: 6519 (49.5%) | 4 y | SSB: cola, squash & sunny delight<br>ASB: diet cola, sugar-free squash<br>Amount: Weekly consumption = 1–6 days a week or Daily consumption = once a day or more than once a day vs less than once a week/never<br>Dietary assessment: questionnaire | Δ BMI,<br>FU BMI,<br>Δ %body fat | Statistical model: linear regression<br>Covariates: age, sex, ethnicity, equivalised household income, maternal education, country, fruit intake, breakfast consumption, physical activity, TV viewing time, & mode of transport to school. | Weekly SSB consumption at 11 y was associated with increases in BMI between ages 7 and 11 (+0.20 kg/m², 95% CI: 0.10, 0.31).<br>Daily SSB consumption at 11 y was associated with increases in BMI between ages 7 and 11 (+0.22 kg/m², 95% CI: 0.11, 0.34).<br>Weekly SSB consumption at 11 y was associated with increases in %body fat between ages 7 and 11 (+0.45%, 95% CI: 0.21, 0.69).<br>Daily SSB consumption at 11 y was associated with increases in %body fat between ages 7 and 11 (+0.57 %, 95 % CI: 0.30, 0.83).<br>Daily ASB consumption at 11 y was associated with increases in BMI between ages 7 and 11 (+0.17 kg/m², 95% CI: 0.06, 0.28). |

| | | | | | | | | | | | |
|---|---|---|---|---|---|---|---|---|---|---|---|
| | | | | | | | | | | | Daily ASB consumption at 11 y was associated with increased %body fat between ages 7 and 11 (+0.35 kg/m$^2$, 95 % CI: 0.09, 0.61). |
| Macintyre et al, 2018 | Growing Up in Scotland (GUS) | Scotland | General population | 2,986<br>5,217 at BL<br>2,986 (57.2%) analysed with complete data | 4-5 y at BL;<br>7-8 y at FU | M: 1526 (51.1%)<br>F: 1460 (48.9%) | 3 y | SSB: soft drinks, diluted juice but not fresh fruit juice<br>ASB: drink diet, low calorie soft drinks, diet or low-calorie flavoured water<br>Amount: higher (At least once a day) or moderate intake (1-6 times/ week) vs lower (<once per week/never)<br>Dietary assessment: questionnaire. | FU OV/OB,<br>FU BMI | Statistical model: multivariate logistic or linear regression models<br>Covariates: self-reported income, maternal education, area-level deprivation, dietary factors (breakfast, milk, water, fruit/vegetables, sweets/crisps, processed meals), TV viewing time, physical activity, BL BMI, & maternal BMI. | No associations were observed between ASB or SSB consumption and risk of OV/OB at 7–8 y.<br>Higher SSB consumption was associated with higher BMI (0.19; 95% CI: 0.01, 0.37; p=0.04), whereas no associations were observed for ASB. |
| Marshall et al, 2019 | Lowa Fluoride Study & Iowa Bone Development<br>Study | USA | General population | 623<br>720 at BL<br>623 (86.5%) analysed with complete data | 2 y | M: 305 (49%)<br>F: 318 (51%) | 15 y | SSB<br>100% juice<br>Milk<br>Water/other sugar-free beverages<br>Amount: 8 oz of additional daily beverage intake<br>Dietary assessment: 3-day validated | FU BMI z score | Statistical model: linear mixed models<br>Covariates: BL socioeconomic status, energy intakes, & mean adequacy ratios | Each additional 8 oz of SSB consumed per day increased the BMI z-score by an average of 0.050 units (CI: 0.022, 0.079; p=0.001).<br>Water/SFB intake was modestly associated with BMI z-score (0.026 units; CI: 0.006, 0.046; p=0.013), while 100% juice (−0.001 units; CI: −0.059, 0.057; p=0.97) and milk (0.022 units; CI: −0.007, 0.052; p=0.13) intakes were not |

| | | | | | | | | | | | |
|---|---|---|---|---|---|---|---|---|---|---|---|
| | | | | | | | | food and beverage diary | | | associated with BMI z-scores. |
| McGovern et al, 2022 | Project Viva, an ongoing prebirth, Boston-area prospective cohort | USA | Clinic (mothers) | 796<br>2,128 at BL<br>796 analysed excluding 1332 (62.5%) those with missing data and dropouts | 3.2±0.2 y at BL;<br>13.1±0.9 y at FU | M: 392 (49.2%)<br>F: 404 (50.8%) | 10 y | Cow milk: whole (3.25%) or 2% milk, lower fat: 1% or skim milk, chocolate milk<br>Amount: higher-fat vs lower-fat milk & Milk frequency (per time/day)<br>Dietary assessment: FFQ | FU OV/OB,<br>FU BMI z score,<br>FU WC,<br>FU % body fat,<br>FU lean mass index,<br>FU sum of subscapular and triceps skinfolds,<br>FU FMI,<br>FU trunk FMI.<br><br>MUO: FU adiponectin (ug/mL), HDL cholesterol (mg/dL), HOMA−IR (units), leptin (ng/mL), triglycerides (mg/dL), metabolic risk z−score (units), systolic blood pressure z−score (units) | Statistical model: multivariable logistic regression models<br><br>Covariates: mutual adjustment for milk fat content and intake frequency, age, parental education & BMI, maternal characteristics (age, marital status, diet, gestational weight gain, parity), socioeconomic factors (household and neighbourhood income), child perinatal factors (age, gestational age, birth weight z-score, breastfeeding, race/ethnicity), early-life behaviours (sleep, TV, diet quality), dietary intakes (energy, SSBs, fiber, juice, sucrose, other dairy), early childhood BMI z-score, & BMI z-score change. | Consumption of whole or 2% milk in early childhood, compared with 1% or skim milk, was associated with lower odds of OV/OB in early adolescence (OR, 0.60; 95% CI, 0.38, 0.93), whereas no association was observed for milk intake frequency.<br>No significant associations were observed between early childhood milk fat intake and adolescent adiposity after full adjustment, although inverse trends were noted in partially adjusted models.<br>Milk intake frequency was not associated with adiposity.<br>Among children with OV/OB, higher-fat milk intake in early childhood was associated with more favourable cardiometabolic markers, including lower HOMA-IR (−0.27; 95% CI: −0.53, −0.02) and higher adiponectin (0.24; 95% CI: 0.05, 0.44). |
| Moore et al, 2023 | National Growth and | USA | Clinics | 2,165<br>2,379 at BL | 9-10 y at BL; | F: 2,165 (100%) | 10 y | Fruit juice: 100% fruit juice | FU BMI | Statistical model: logistic regression models | Girls consuming ≥1.25 cups/day of fruit juice had a BMI in late adolescence that |

| | | | | | | | | | | |
|---|---|---|---|---|---|---|---|---|---|---|
| | Health Study (NGHS) | | | 2,165 analysed after excluding 214 (8.9%) | 17-20 y at FU | | | (consumed as a beverage)<br>Amount: ≥1.25 cups vs 0 cups<br>Dietary assessment: 3-day dietary records | | Covariates: age, race, socioeconomic status (parental education), physical activity, BL BMI, total energy intake, & % energy from carbohydrates, protein, & fat. | was 1.7 kg/m$^2$ lower than that of non-juice-drinking girls (p=0.0022). |
| Sakaki et al, 2021 | Growing Up Today Study II (GUTS) | USA | General population & community | 7,301<br>10,918 at BL<br>7,301 analysed excluding 33.1% with missing data | 13.4±1.8 y for boys, 13.5±1.8 y for girls at BL | M: 4,744 (45%)<br>F: 5,817 (55%) | 2 y | Orange juice<br>Amount: never/less than 1 per month, 1–3 glasses per month, 1–6 glasses per week, and ≥ 1 glass per day<br>Dietary assessment: semi-quantitative FFQ | Δ BMI,<br>Δ BMI percentile | Statistical model: mixed linear regression models<br>Covariates: age, race, BL BMI percentile, 2-year changes in total energy intake excluding orange juice, physical activity, & screen time. | Orange juice consumption was not associated with 2-year change in BMI and BMI percentile. |
| Scharf et al, 2013 | Early Childhood Longitudinal Survey – Birth (ECLS-B) | USA | General population | 8,300<br>10,700 at BL<br>8,300 analysed with complete data after removing missing data (22.5%) | 2 y at BL;<br>4 y at FU | M: 4200 (51.1%)<br>F: 4100 (48.8%) | 2 y | Types of milk: 1%/skim compared to whole/2%<br>Amount: Not specified (categorical comparison of milk type)<br>Dietary assessment: questionnaire. | OV/OB,<br>Δ BMI z-score | Statistical model: multivariable linear regression models<br>Covariates: sex, race, socioeconomic status, juice & SSB intake, number of glasses of milk daily, & maternal BMI. | Across racial/ethnic and SES subgroups, 1%/skim-milk drinkers had higher BMI z-scores than 2%/whole-milk drinkers. In multivariable analyses, increasing fat content in the type of milk consumed was inversely associated with BMI z-score (p<0.0001).<br>Among children who were normal weight at BL, consistent intake of 1%/skim milk compare to 2%/whole was associated with increased odds of becoming OV/OB between 2 and 4 |

| | | | | | | | | | | | |
|---|---|---|---|---|---|---|---|---|---|---|---|
| | | | | | | | | | | | years (OR, 1.57; 95% CI: 1.03, 2.42, p=0.04).<br>Consumption of 1%/skim milk at 2 was not associated with change in BMI z-score over time (p=0.6). |
| Shefferly et al, 2017 | Early Childhood Longitudinal Study–Birth (ECLS-B) | USA | General population | 8,950<br>10,700 at BL<br>8,950 (83.6%) analysed with complete FU data | 2 y at BL;<br>4 y at FU | M: 4582 (51.2%)<br>F: 4368 (48.8%) | 2 y | 100% fruit juice: orange, apple, or grape juice. Exclude punch, Sunny Delight, Kool-Aid, sports drinks, or other fruit-flavoured drinks.<br>Amount: Serving 8 oz.<br>≥1 serving daily vs <1 serving daily<br>Dietary assessment: questionnaire. | OV/OB,<br>Δ BMI z-score | Statistical model: linear and logistic multivariable regression models<br>Covariates: sex, race/ethnicity, socioeconomic status, maternal BMI, & BL BMI z-score. | Among children with normal weight at 2 years, consistent juice consumption was associated with increased odds of becoming OV (OR, 1.30; 95% CI: 1.06, 1.59; p=0.0129), with no association observed for OB.<br>Juice consumption at 2 years was associated with a greater increase in BMI z-score between ages 2 and 4 years compared with infrequent/non-consumers (0.28 vs 0.03 units; p=0.0003). |
| Zheng et al, 2014 | European Youth Heart Study (EYHS) | Denmark | Clinical | 283<br>590 at BL<br>283 analysed after removing 281 dropouts, 25 incomplete data and 1 (52%) underreporting. | 9.6±0.4 y at BL | M: 125 (44%)<br>F: 158 (56%) | 6 & 12 y | SSB: regular soft drinks, fruit drinks and cordials sweetened with caloric sweeteners but excluded 100% fruit juice, flavoured milk, coffee and tea.<br>Amount: >1 serve per day, ≤1 serve per day, & non-consumer. | Δ BMI,<br>Δ WC,<br>Δ S4SF<br>from 9–21 y & from 15–21 y | Statistical model: multivariate regression model<br>Covariates: change in SSB consumption and ΔBMI/ΔWC/ΔS4SF; age), sex, BMI/ WC/ sum of four skinfolds (15 y), SSB intake (9 y), socioeconomic status, pubertal status, physical activity (15 y), change in energy intake from ages 9–15 y, change in HOMA-IR from ages 9–15 y. | SSB consumption at 9 years was not associated with changes in BMI or WC between ages 15 and 21 years.<br>SSB consumption >1 serving/day at 15 years was associated with greater increases in BMI (β, 0.92; p=0.046) and WC (β, 2.69; p=0.04) between ages 15 and 21 years compared with non-consumers, although associations were attenuated |

| | | | | | | | | | | | |
|---|---|---|---|---|---|---|---|---|---|---|---|
| | | | | | | | | Dietary assessment: 24h recall & qualitative food record at 9 and 15 y | | | after further adjustment for energy intake or HOMA-IR.<br>Increasing SSB consumption between ages 9 and 15 years was associated with greater increases in WC (β, 2.72; p=0.04) and a non-significant increase in BMI (β, 0.91; p=0.09) between ages 15 and 21 years.<br>No significant associations were observed for changes in skinfold thickness. |
| Zheng et al, 2015 | Childhood Asthma Prevention Study (CAPS) | Australia | Population | 158<br>237 at BL<br>158 (66.6%) analysed with complete data at FU | 8.0±0.2 y at BL;<br>11.5±0.2 y at FU | M:82 (51.8%)<br>F: 76 (48.2%) | 3.5 y | Water: tap, bottled & unflavoured mineral,<br>SSB: regular soft drinks, fruit drinks, cordials & sugar-sweetened sport drinks,<br>Milk: full fat, reduced fat, skim & flavoured,<br>Coffee/tea: plain & sweetened,<br>100% fruit juice: apple, blackcurrant, grape, orange & fruit blend,<br>Diet drink: low energy drinks sweetened with | Δ BMI z-score<br>FU % body fat | Statistical model: multivariate linear regression models<br>Covariates: age, gender, BMI z-score at age 8 y, Socioeconomic Index for Area scores, maternal age at birth, parental education level, parental countries of birth, maternal age at birth, presence of gestational diabetes, breastfeeding characteristics, pubertal status, Childhood Asthma Prevention Study randomisation group, & total energy intake. | SSB intake at 9 years was associated with greater increases in Δ BMI z-score (β: 0.10; p=0.003) and % body fat (β: 1.04%; p=0.001), with dose–response relationships observed across intake quartiles (p-trend ≤0.02).<br>Diet drink consumption was inversely associated with Δ BMI z -score (β: -0.20; p=0.01) and % body fat (β: -1.41; p=0.046) after adjustment for total energy intake, whereas no significant associations were observed for other beverage types. |

| | | | | | | | | | | | |
|---|---|---|---|---|---|---|---|---|---|---|---|
| | | | | | | | | artificial sweeteners.<br>Amount: 100 g/day<br>Dietary assessment: 3 24h dietary recall at 9 y | | | |
| Zheng et al, 2015 | Healthy Start Study | Denmark | Community & general population | 352<br>552 at BL (186 (33.6%) drop-out; 14 (2.5%) exclude)<br>352 analysed | 4.1±1.1 y at BL;<br>5.4±1.1 y at FU | M: 195 (55%)<br>F: 158 (45%) | 1.5 y | Water: tap water, sparkling water & still water,<br>Milk: skimmed milk, low-fat milk, whole milk, butter milk & flavoured milk,<br>Sugary drinks: sugar-sweetened carbonated & fruit-flavoured drinks<br>Fruit juice,<br>Diet drinks: artificially sweetened beverages.<br>Amount: g/day<br>Dietary assessment: 4-d dietary record | Δ BMI z-score | Statistical model: multivariable linear regression<br>Covariates: BL age, BMI z-score, sex, intervention group, physical activity, family structure (parental divorce, siblings), socioeconomic factors (income, parental education), and maternal pre-pregnancy OV, total energy intake (nutrient residual model) & non-beverage energy intake (energy partition model). | Sugary drink intake at BL was associated with greater increases in BMI z-score (β=0.06; p=0.04) over 1.5 y; however, these associations were attenuated after adjustment for total energy intake. Results remained similar in energy partition models.<br>No associations were observed for other beverage types, and changes in beverage intake were not associated with changes in BMI z-score. |

[a] Presented as mean ± standard deviation and/or range. [b] Presented as absolute (relative) frequency.

Abbreviations: ASB: artificially sweetened beverage; BL: baseline; BMI: body mass index; CI: confidence interval; d: day(s); DXA: dual-energy X-ray absorptiometry; F: females; FMI: fat mass index; FU: follow-up; h: hour(s); HDL: high-density lipoprotein; HOMA-IR: homeostasis model assessment – insulin resistance; M: males; NR: not

reported; OB: obesity; OV: overweight; RCT: randomized controlled trial; S4SF: sum of 4 skinfolds; SSB: sugar-sweetened beverage; TBFM: total body fat mass; TV: television; UPF: ultra-processed foods; WC: waist circumference; y: year(s); Δ: change.

**Supplementary Table S4. Overview of RCTs examining the effect of interventions targeting foods/ food groups on the risk of childhood OV/OB.**

| Study | | Country | Setting | Population | | | FU | Interventions | Outcomes | Statistical analysis | Results |
|---|---|---|---|---|---|---|---|---|---|---|---|
| Author, year | Title (acronym) | | | N | Age [a] | Sex [b] | | | | | |
| Bere et al, 2014 | Fruits and Vegetables Make the Marks project (FVMM) | Norway | School | 320<br>1950 at BL<br>1602 in 2005; 320 in 2009 (84.6% drop-out)<br>320 (16.4%) analysed | 11.8 ± 0.2 y<br>10-12 y | M: 984 (50.5%)<br>F: 966 (49.5%) | 7 y | Design: Cluster RCT<br>Duration: 9 mo<br>Groups: IG vs CG<br>IG (n=585): Free fruit (1 piece/day) for 9 mo without payment.<br>CG (n=1365): No intervention.<br>Dietary assessment: 24-hour recall (portions/day) and food frequency questionnaire | OV,<br>BMI | Statistical model: multivariate logistic regression (OR, 95%CI, p-value)<br>Covariates: school, sex, grade level, parental education | At 4-year follow-up, OV prevalence was lower in the IG compared with CG (15% vs 25%), with an unadjusted OR of 0.52 (95% CI: 0.28–0.97). However, this association was not statistically significant after adjustment for covariates and clustering. No significant differences were observed for BMI between groups. |
| Katan et al, 2016 | Double-blind Randomized INtervention study in Kids (DRINK) | Amsterdam | School | 477<br>641 at BL<br>48 dropouts in 6 mo<br>40 dropouts in 12 mo<br>477 (74.4%) with complete FU analysed | 8.2±1.9 y<br>5-11 y | M: 270 (57%)<br>F: 207 (43%) | 18 mo | Design: Double-blind RCT<br>Duration: 18 mo<br>Groups: IG vs CG<br>IG (n=225): Daily replacement of sugar-sweetened beverages with identical-looking, non-caloric artificially sweetened beverages (~250 mL/day)<br>CG (n=252): Continued consumption of sugar-sweetened beverages (control condition)<br>Dietary assessment: Not formally assessed; adherence evaluated | Body weight,<br>BMI z-score,<br>FM,<br>Sum of four skinfolds,<br>FM | Statistical model: multivariable linear regression with interaction term (effect modification by baseline BMI)<br>Covariates: age, sex, parental education, ethnicity | Children receiving sugar-free beverages had a smaller increase in BMI z-score compared with those receiving sugar-sweetened beverages, with a stronger effect among those with higher baseline BMI (−0.21 SD vs −0.05 SD). The difference in effect between BMI groups was −0.16 SD (95% CI: −0.31 to −0.01; p=0.04).<br>Body weight gain was reduced by 1.53 kg in the higher BMI group and 0.62 kg in the lower BMI group (interaction p=0.09).<br>Similar patterns were observed for WC, skinfold |

| | | | | | | | | | | | |
|---|---|---|---|---|---|---|---|---|---|---|---|
| | | | | | | | | through recorded consumption of provided beverages | | | thickness, and FM, with greater reductions among children with higher baseline BMI. No substantial changes were observed after adjustment for covariates. |
| Rifas-Shiman et al, 2017 | High Five for Kids | USA | Primary care | 441<br>475 at BL (34 (7%) drop-out<br>441 analysed | 4.9± 1.2 y | M: 228 (51.7%)<br>F: 213 (48.3%) | 1 y (BL to mid intervention)<br>2 y (BL to end of intervention) | Design: Cluster RCT<br>Duration: 2 y<br>Groups: IG vs CG<br>IG (n=249): 1-year intensive behavioural intervention (4 in-person visits, 2 phone calls) followed by 1-year maintenance (2 visits), using motivational interviewing targeting reduction of fast food and sugar-sweetened beverage intake<br>CG (n=192): Usual care<br>Dietary assessment: questionnaire. | BMI z-score | Statistical model: multivariable linear regression<br>Covariates: age, sex, race/ethnicity, parental education, parental OV/OB, household income | BMI increased in both groups, but BMI z-scores decreased (−0.20 in IG vs −0.18 in CG), with no significant between-group difference (adjusted Δ −0.04; 95% CI: −0.14 to 0.06). |

[a] Presented as mean ± standard deviation and/or range. [b] Presented as absolute (relative) frequency.

Abbreviations: BL: baseline, BMI: body mass index, CG: control group, CI: confidence interval, d: day(s), F: females, FM: fat mass, FU: follow-up, IG: intervention group, M: males, mo: month(s), NR: not reported, OB: obesity, OV: overweight, RCT: randomised controlled trial, y: year(s), Δ: change.
